# Barriers to care for people with unclear visual loss—Data from a tertiary-level-of-care neuroinflammation center

**DOI:** 10.1177/20552173251397772

**Published:** 2025-12-05

**Authors:** Murat Delikaya, Charlotte Bereuter, Jan Schroeter, Elisa Nowak, Eva-Maria Dorsch, Lidia Kilinska, Joseph Kuchling, Nadja Siebert, Janina Behrens, Friedemann Paul, Judith Bellmann-Strobl, Tanja Schmitz-Hübsch, Frederike Cosima Oertel

**Affiliations:** Experimental and Clinical Research Center, 196150 Max Delbrueck Center for Molecular Medicine and Charité – Universitätsmedizin Berlin, Corporate Member of Freie Universität Berlin and Humboldt-Universität zu Berlin, Berlin, Germany; Neuroscience Clinical Research Center, 14903 Charité – Universitätsmedizin Berlin, Corporate Member of Freie Universität Berlin and Humboldt-Universität zu Berlin, Berlin, Germany; Department of Neurology, 14903 Charité – Universitätsmedizin Berlin, Corporate Member of Freie Universität Berlin and Humboldt-Universität zu Berlin, Berlin, Germany; Experimental and Clinical Research Center, Max Delbrueck Center for Molecular Medicine and Charité – Universitätsmedizin Berlin, Corporate Member of Freie Universität Berlin and Humboldt-Universität zu Berlin, Berlin, Germany; Neuroscience Clinical Research Center, 14903 Charité – Universitätsmedizin Berlin, Corporate Member of Freie Universität Berlin and Humboldt-Universität zu Berlin, Berlin, Germany; Experimental and Clinical Research Center, Max Delbrueck Center for Molecular Medicine and Charité – Universitätsmedizin Berlin, Corporate Member of Freie Universität Berlin and Humboldt-Universität zu Berlin, Berlin, Germany; Experimental and Clinical Research Center, Max Delbrueck Center for Molecular Medicine and Charité – Universitätsmedizin Berlin, Corporate Member of Freie Universität Berlin and Humboldt-Universität zu Berlin, Berlin, Germany; Neuroscience Clinical Research Center, 14903 Charité – Universitätsmedizin Berlin, Corporate Member of Freie Universität Berlin and Humboldt-Universität zu Berlin, Berlin, Germany; Experimental and Clinical Research Center, Max Delbrueck Center for Molecular Medicine and Charité – Universitätsmedizin Berlin, Corporate Member of Freie Universität Berlin and Humboldt-Universität zu Berlin, Berlin, Germany; Neuroscience Clinical Research Center, Charité – Universitätsmedizin Berlin, Corporate Member of Freie Universität Berlin and Humboldt-Universität zu Berlin, Berlin, Germany; Department of Neurology, Charité – Universitätsmedizin Berlin, Corporate Member of Freie Universität Berlin and Humboldt-Universität zu Berlin, Berlin, Germany; Experimental and Clinical Research Center, Max Delbrueck Center for Molecular Medicine and Charité – Universitätsmedizin Berlin, Corporate Member of Freie Universität Berlin and Humboldt-Universität zu Berlin, Berlin, Germany; Neuroscience Clinical Research Center, Charité – Universitätsmedizin Berlin, Corporate Member of Freie Universität Berlin and Humboldt-Universität zu Berlin, Berlin, Germany; Experimental and Clinical Research Center, Max Delbrueck Center for Molecular Medicine and Charité – Universitätsmedizin Berlin, Corporate Member of Freie Universität Berlin and Humboldt-Universität zu Berlin, Berlin, Germany; Neuroscience Clinical Research Center, Charité – Universitätsmedizin Berlin, Corporate Member of Freie Universität Berlin and Humboldt-Universität zu Berlin, Berlin, Germany; Department of Neurology, Charité – Universitätsmedizin Berlin, Corporate Member of Freie Universität Berlin and Humboldt-Universität zu Berlin, Berlin, Germany; Experimental and Clinical Research Center, Max Delbrueck Center for Molecular Medicine and Charité – Universitätsmedizin Berlin, Corporate Member of Freie Universität Berlin and Humboldt-Universität zu Berlin, Berlin, Germany; Neuroscience Clinical Research Center, Charité – Universitätsmedizin Berlin, Corporate Member of Freie Universität Berlin and Humboldt-Universität zu Berlin, Berlin, Germany; Experimental and Clinical Research Center, Max Delbrueck Center for Molecular Medicine and Charité – Universitätsmedizin Berlin, Corporate Member of Freie Universität Berlin and Humboldt-Universität zu Berlin, Berlin, Germany; Neuroscience Clinical Research Center, Charité – Universitätsmedizin Berlin, Corporate Member of Freie Universität Berlin and Humboldt-Universität zu Berlin, Berlin, Germany; Department of Neurology, Charité – Universitätsmedizin Berlin, Corporate Member of Freie Universität Berlin and Humboldt-Universität zu Berlin, Berlin, Germany; Experimental and Clinical Research Center, Max Delbrueck Center for Molecular Medicine and Charité – Universitätsmedizin Berlin, Corporate Member of Freie Universität Berlin and Humboldt-Universität zu Berlin, Berlin, Germany; Neuroscience Clinical Research Center, Charité – Universitätsmedizin Berlin, Corporate Member of Freie Universität Berlin and Humboldt-Universität zu Berlin, Berlin, Germany; Experimental and Clinical Research Center, Max Delbrueck Center for Molecular Medicine and Charité – Universitätsmedizin Berlin, Corporate Member of Freie Universität Berlin and Humboldt-Universität zu Berlin, Berlin, Germany; Neuroscience Clinical Research Center, Charité – Universitätsmedizin Berlin, Corporate Member of Freie Universität Berlin and Humboldt-Universität zu Berlin, Berlin, Germany; Department of Neurology, Charité – Universitätsmedizin Berlin, Corporate Member of Freie Universität Berlin and Humboldt-Universität zu Berlin, Berlin, Germany; Einstein Center Digital Future, Berlin, Germany

**Keywords:** Barriers in healthcare, vision loss, neurovisual impairment, multiple sclerosis, NMOSD

## Abstract

**Background:**

Visual symptoms are common in people with multiple sclerosis. The revised 2024 McDonald criteria include the optic nerve as a fifth anatomical region, underscoring the need for specific diagnostics. Although optical coherence tomography (OCT) and visual evoked potentials (VEP) are available, the extent of their routine pre-referral use is insufficiently documented. We evaluated pre-referral utilization and hypothesized that specific diagnostics are used less often than non-specific diagnostics and that differences are not explained by demographics alone.

**Methods:**

Retrospective cross-sectional study of 305 patients referred for visual symptoms to a tertiary neuroimmunology clinic in Germany. Analyses focused on people with multiple sclerosis (*n* = 112) and disease controls with neuromyelitis optica spectrum disorders or myelin oligodendrocyte glycoprotein-associated disease (pwNM; *n* = 36).

**Results:**

In people with multiple sclerosis, only 6.2% received OCT and 33% VEP for their visual complaints, compared to unspecific diagnostics such as cranial magnetic resonance imaging (58%) and lumbar puncture (42%) – independent of demographic factors.

**Conclusion:**

The pre-referral use of specific neurovisual tests in people with multiple sclerosis with visual symptoms was low relative to non-specific procedures. This suggests heterogeneous integration of neurovisual testing across care levels. In light of the revised McDonald Criteria 2024, prospective multicenter studies should examine implementation and clinical impact.

## Introduction

Visual complaints are among the most common symptoms in people with multiple sclerosis (pwMS)^1^ and can substantially impair the quality of life.^2,3^ Visual complaints in pwMS can arise from different mechanisms. On the one hand, they include symptoms such as blurred vision, double vision, or visual field loss. On the other hand, they may reflect clinically defined syndromes such as optic neuritis, internuclear ophthalmoplegia, or uveitis, each representing distinct forms of visual pathway involvement. In addition, higher-order cortical processing can contribute to visual dysfunction, further illustrating the heterogeneity of visual problems in multiple sclerosis (MS).^4–7^

Despite their high clinical relevance, visual complaints often receive less attention in the context of MS care than motor or cognitive deficits.^8^ Routine neurological diagnostics, such as neurological examination and Snellen eye chart test, are often not sufficiently sensitive for the detection of subtle or chronic visual system involvement. Established diagnostic tools for assessing the visual pathway, such as optical coherence tomography (OCT),^9^ visual evoked potentials (VEP),^10^ and visual field tests, are common procedures that enable an objective functional and structural assessment of the visual system,^11^ but their utilization varies across settings and health systems, shaped by local availability and clinical workflows.

The addition of the optic nerve as the fifth criterion for dissemination in space in the revision of the 2024 McDonald criteria for MS emphasizes the increasing significance of objectively detecting optic nerve involvement during neurological assessments in pwMS.^12,13^ However, the structure of access to diagnostic procedures that can reliably objectify such involvement has not been investigated. While VEP and visual field testing are recognized procedures for neurological diagnostics, there is a lack of reliable data regarding their application in routine clinical practice worldwide — and in Germany — to date. Access to these diagnostic examinations, particularly OCT, is influenced by the structures and incentive mechanisms within the respective healthcare systems. To date, there has been no systematic investigation of the care situation of pwMS with visual complaints and influencing factors on the level of care.

Thus, the aim of this study was to evaluate pre-referral diagnostic care utilization for pwMS with visual complaints in a single tertiary neuroimmunology clinic in Germany. We further examined whether these patterns varied across demographic and clinical subgroups. We hypothesized that specific diagnostics (OCT, VEP and visual fields) would be less frequently documented than non-specific diagnostics (cranial magnetic resonance imaging [cMRI], lumbar puncture and blood sample), and that this imbalance would persist across strata of age, sex, symptom duration and accompanying symptoms. All analyses were descriptive and intended to characterize utilization patterns rather than assess appropriateness or causality. Descriptive data from the total referral cohort are presented to provide a broader healthcare context, while primary analyses focus on pwMS.

## Methods

### Ethics approval

The institutional ethics committee of Charité – Universitätsmedizin Berlin approved the study (EA1/266/23), and the study was carried out in accordance with the Declaration of Helsinki (1160/2017). The ethics committee waived the requirement for participants’ formal informed consent as this was a retrospective study, and no individual patient-level data were reported.

Data from this project are not publicly available due to a lack of patient consent.

### Study design and population

This single-center retrospective cross-sectional study was conducted in the neurovisual outpatient clinic of the Neuroimmunology Department at Charité – Universitätsmedizin Berlin. The clinic provides specialized assessment for adult patients with complex or unclear visual symptoms in the context of suspected neuroinflammatory or autoimmune diseases, often after initial workups in other settings. Patients are referred by neurologists, ophthalmologists, or general practitioners — either for differential diagnostic clarification or for further evaluation and management of known conditions with visual involvement.

We included all adult patients who presented between January 2015 and August 2023 with visual symptoms (e.g. eye movement pain, visual field deficits and double vision). Patients without visual symptoms — that is, those referred solely for differential diagnostic workup of systemic inflammatory diseases without visual complaints — were excluded.

**Figure 1.**
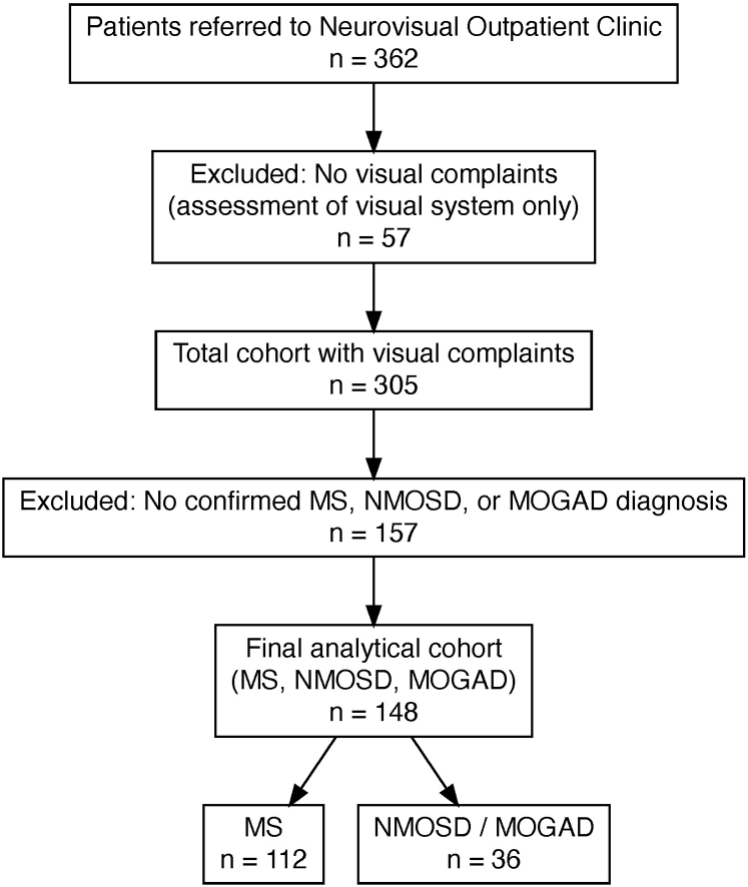
Flowchart illustrating the selection of the study cohort. Of 362 patients referred to the Neurovisual Outpatient Clinic, 57 were excluded because they had no visual symptoms and were referred only for visual system assessment. The remaining 305 patients presented with visual complaints. Of these, 157 were not included in the final cohorts because no diagnosis of MS, NMOSD, or MOGAD was established. The final cohort consisted of 148 people with confirmed diagnoses of MS (pwMS; *n* = 112) and NMOSD/MOGAD as controls (pwNM; *n* = 36). MS: multiple sclerosis; NMOSD: neuromyelitis optica spectrum disorders; MOGAD: myelin oligodendrocyte glycoprotein antibody-associated disease; pwMS: people with multiple sclerosis.

The total cohort (*N* = 305) included patients with neuroinflammatory, rheumatological, and ophthalmological conditions (Figure 1). For our primary analyses, we included only those patients from the total cohort with a confirmed diagnosis of MS (pwMS; *n* = 112) or with neuromyelitis optica spectrum disorder/myelin oligodendrocyte glycoprotein antibody-associated disease (pwNM; *n* = 36). The pwNM group served as disease controls, as they presented with similar visual symptoms and also required specific diagnostics, but differed from pwMS in prevalence and clinical management. Diagnoses were made by the referring or treating physicians according to the internationally accepted diagnostic criteria applicable at the time of presentation. Our study did not reassign diagnoses.^14–17^

### Clinical data collection

The dataset was created from digital and non-digital patient records and entered into a REDCap-based questionnaire.^18^ Clinical and diagnostic history was extracted retrospectively from routine clinical documentation. No standardized questionnaire or structured symptom checklist was used at the time of data collection. Parameters were collected in the following categories: demographics, referral details, medical history and prior diagnostic workup in relation to their visual complaints and in-house neurovisual assessments. Referral data included information on the specialty of referral (internal or external) and the reasons for referral (e.g., visual loss, visual field loss and nystagmus). Medical history data included all medical conditions and duration of the visual complaints (<1 month, 1–12 months, 1–5 years and > 5 years) and the affected eyes. Diagnostic workup data included a systematic documentation of prior examinations performed in connection with the visual complaints at any time prior to the first presentation, provided they were clearly related to the reported visual complaints, such as VEP, OCT, visual field testing, cMRI, lumbar puncture and standard laboratory workup (blood samples). Barriers were defined as the under-use of specific diagnostics compared with non-specific diagnostics and assessed by comparing utilization rates across subgroups.

As part of their outpatient visit, all patients received a comprehensive neurovisual diagnostic workup (including as relevant OCT, VEP, visual field testing, measurement of intraocular pressure, measurement of high-contrast (HC-VA) and low-contrast visual acuity (LC-VA)). Test results were abstracted from routine medical records and coded from the examining physician's report. Results were coded as “non-normal” if interpreted as abnormal (e.g. below specific thresholds). If no interpretation was available or documentation was inconclusive, results were coded as “not evaluable.” Raw data were not reanalyzed. Information on neurological examinations was extracted from routine records and examinations were not performed for study purposes. Whether an examination was performed was at the treating physician's discretion, for example, in the presence of acute focal deficits, unclear or progressive symptoms, or for triage. We coded only performance, yes/no, and the contemporaneous findings. The compiler did not reinterpret results. This variable documented available clinical information and was not included in the primary analyses focused on pre-referral utilization.

### Statistical methods

Groups were separated by diagnosis (total cohort, pwMS, and pwNM), duration of visual symptoms (<1 year vs. ≥ 1 year), presence of accompanying symptoms (yes vs. no), sex and age group (categorized by decade). We included sex and age as they may influence diagnostic utilization.^19,20^ Accompanying symptoms were defined as symptoms that were reported in addition to the neurovisual complaints during the neurovisual consultation, including, for example, depressive symptoms, fatigue, sensory disturbances, nausea, headaches and dizziness.

Within each diagnostic group, we compared the proportions of patients who received specific diagnostics across subgroups: If not stated otherwise, continuous numerical variables (e.g., age) are reported as mean *±* standard deviation (SD) and compared between groups using *t*-tests. Categorical variables (e.g. sex, referral source and diagnostic results) are reported as absolute and relative frequencies and compared between groups using Chi-square tests or Fisher's exact tests, as appropriate. To assess potential diagnostic gaps prior to the neurovisual consultation, we additionally performed pairwise comparisons between non-specific diagnostics (blood sampling, cMRI, and lumbar puncture) and specific diagnostics (OCT, VEP, and visual field testing). Group differences were calculated as the absolute difference in proportions of patients who had received each diagnostic modality, expressed as:

[% with standard diagnostic procedure] – [% with corresponding neurovisual diagnostic].

These comparisons were conducted separately within the total cohort, pwMS, and pwNM groups. Although the main study focus is on pwMS (with pwNM serving as a comparator group), results are additionally reported for the full total cohort to provide an overview of diagnostic utilization patterns. All comparisons are exploratory and were not adjusted for multiple testing, as the study aimed to describe utilization patterns across several related diagnostic procedures in relatively small subgroups. All statistical analyses were performed using R (Version 2023.09.1) with packages ggplot2 and dplyr.^21^ A *p*-value <0.05 was considered statistically significant. Data from this project are not available due to constraints of the ethical approval.

## Results

### Cohort

In total, 305 patients were included (Table 1). pwMS (*n* = 112) and pwNM (*n* = 36) did not differ in age (*p* = 0.442) or sex (*p* = 0.235). At the time of the visit, most patients had suffered from neurovisual complaints longer than 12 months (total cohort: 62.2%, pwMS: 65.2% and pwNM: 58.3%) and most patients reported accompanying symptoms (total cohort: 68.5%, pwMS: 72.3% and pwNM: 80.6%).

**Table 1.** Demographic data.

Cohort			
	Total cohort	pwMS	pwNM
People [*n*]	305	112	36

Age [years, mean ± SD]	41 ± 14	40 ± 12	40 ± 13
Sex [female, *n* (%)]	206 (68.0)	73 (65.0)	26 (72.0)
**Duration of visual symptoms**			
< 1 month [*n* (%)]	15 (4.9)	7 (6.2)	2 (5.6)
1–12 months [*n* (%)]	99 (32.5)	32 (28.6)	13 (36.1)
1–5 years [*n* (%)]	111 (36.4)	33 (29.5)	7 (19.4)
> 5 years [*n* (%)]	80 (26.2)	40 (35.7)	14 (38.9)
People with accompanying symptoms [*n* (%)]	209 (68.5)	81 (72.3)	29 (80.6)
**Referred by**			
Neurology [*n* (%)]	195 (63.9)	84 (75.0)	25 (69.4)
Ophthalmology [*n* (%)]	29 (9.5)	5 (4.5)	3 (8.3)
Others or not known [*n* (%)]	81 (26.6)	23 (20.5)	8 (22.3)
**Prior diagnostic work-up related to visual complaints**			
People with prior diagnostics [total, *n* (%)]	252 (82.6)	78 (69.6)	32 (88.9)
Prior Blood sample [*n (%)*]	133 (43.6)	37 (33.0)	26 (72.2)
Prior cMRI [*n (%)*]	219 (71.8)	65 (58.0)	24 (66.7)
Prior Lumbar puncture [*n (%)*]	148 (48.5)	47 (42.0)	19 (52.8)
Prior OCT [*n* (%)]	34 (11.1)	7 (6.2)	6 (16.7)
Prior VEP [*n* (%)]	124 (40.7)	37 (33.0)	18 (50.0)
Prior Visual Field [*n* (%)]	27 (8.9)	5 (4.5)	1 (2.8)

pwMS: people with multiple sclerosis; pwNM: people with NMOSD or MOGAD; NMOSD, neuromyelitis optica spectrum disorder; MOGAD: myelin oligodendrocyte glycoprotein antibody-associated disease; cMRI: cranial magnetic resonance imaging; OCT: optical coherence tomography; VEP: visual evoked potentials.

### Prior diagnostic work-up related to visual complaints

#### Diagnostic workup

**Figure 2.**
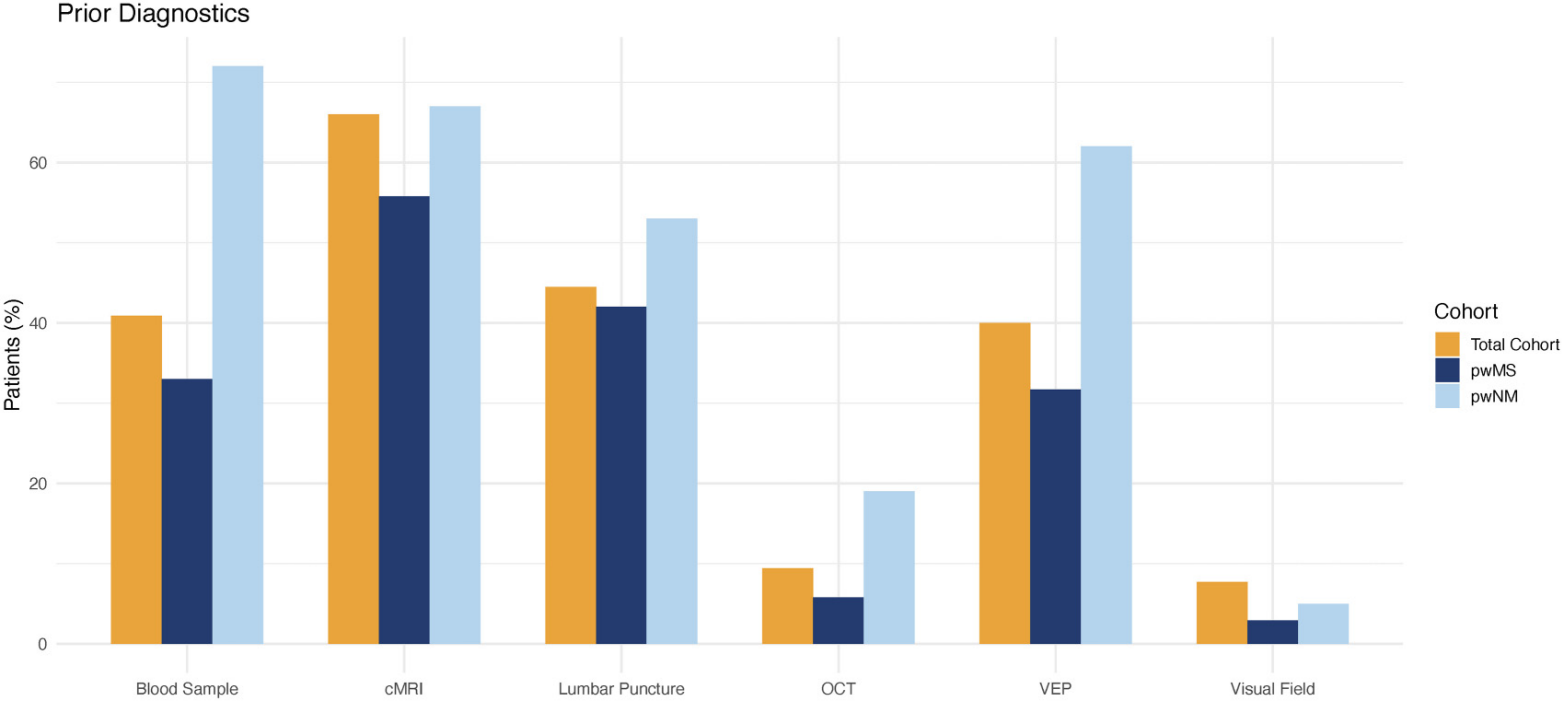
Frequency of prior examinations by cohort for the total cohort (yellow), pwMS (dark blue) and pwNM (light blue). MRI: magnetic resonance imaging; VEP: visual evoked potentials; OCT: optical coherence tomography; pwMS: people with multiple sclerosis; pwNM: people with neuromyelitis optica spectrum disorders and myelin oligodendrocyte glycoprotein antibody-associated disease.

Prior to the outpatient visit, most patients had diagnostic procedures for their visual complaints (total cohort: 82.6%, pwMS: 69.6% and pwNM: 88.9%). The most frequently performed diagnostic procedure was cMRI (total cohort: 71.8%, pwMS: 58% and pwNM: 66.7%), followed by lumbar puncture (total cohort: 48.5%, pwMS: 42% and pwNM: 52.8%) and blood samples (total cohort: 43.6%, pwMS: 33% and pwNM: 72.2%). Diagnostic workup more specifically targeted at neurovisual complaints, such as VEP (total cohort: 40.7%, pwMS: 33% and pwNM: 50%), OCT (total cohort: 11.1%, pwMS: 6.2% and pwNM: 16.7%), and visual fields (total cohort: 8.9%, pwMS: 4.5% and pwNM: 2.8%), were less frequently conducted (Table 2). The frequencies of prior diagnostic examination are summarized in Figure 2.

**Table 2.** Pairwise comparisons between standard diagnostic procedures (blood sample, cranial MRI, and lumbar puncture) and neurovisual diagnostics (OCT, VEP, and visual field testing) that had already been performed prior to referral to the neurovisual outpatient clinic.

Prior diagnostic work-up related to visual complaints										
Vs.	Cohort	OCT			VEP			Visual field		
		*p*	95% CI	Group difference [%]	*p*	95% CI	Group difference [%]	*p*	95% CI	Group difference [%]
Blood sample	Total cohort	<0.001	[25.9; 39.1]	32.5	0.461	[−4.9; 10.8]	3.0	<0.001	[28.3; 41.2]	34.8
	Multiple sclerosis	<0.001	[17.0; 36.6]	26.8	0.878	[−12.3; 12.3]	0.0	<0.001	[19.1; 38.1]	28.6
	NMOSD/MOGAD	<0.001	[35.9; 75.2]	55.6	0.375	[−0.4; 44.9]	22.2	<0.001	[53.2; 85.7]	69.4
cMRI	Total cohort	<0.001	[54.5; 66.8 ]	60.7	<0.001	[23.7; 38.6]	31.1	<0.001	[57.0; 68.9]	63.0
	Multiple sclerosis	<0.001	[41.6 ; 62.0]	51.8	<0.001	[12.4; 37.6]	25.0	<0.001	[43.7 ; 63.5]	53.6
	NMOSD/MOGAD	<0.001	[29.7; 70.3]	50.0	0.773	[−6.5; 39.8]	16.7	<0.001	[46.9; 80.9]	63.9
Lumbar puncture	Total cohort	<0.001	[30.7; 44.0 ]	37.4	0.051	[0.0; 15.7]	7.9	<0.001	[33.2; 46.1]	39.7
	Multiple sclerosis	<0.001	[25.5; 45.9]	35.7	0.299	[−3.7; 21.6]	8.9	<0.001	[27.6; 47.4]	37.5
	NMOSD/MOGAD	0.071	[15.1; 57.1]	36.1	0.165	[−21.0; 26.6]	2.8	<0.001	[32.1; 67.9]	50.0

All confidence intervals refer to the reported group differences. Group differences are defined as [% in standard diagnostic group] – [% in neurovisual diagnostic group]. Results are exploratory and not adjusted for multiple testing.

OCT: optical coherence tomography; VEP: visual evoked potentials; *p*: *p*-value; 95% CI: 95% confidence interval; blood sample, cMRI: cranial magnetic resonance imaging; NMOSD, neuromyelitis optica spectrum disorder; MOGAD: myelin oligodendrocyte glycoprotein antibody-associated disease.

**Table 3.** Comparison of demographic and clinical characteristics between pwMS with and without prior diagnostic work-up before presentation to the neurovisual outpatient clinic.

	PwMS with prior diagnostic	PwMS without prior diagnostic	PwMS with prior diagnostic vs. PwMS without prior diagnostic		
People [*n*]	78	34			
			*p*	95% CI	Group difference
Accompanying symptoms [*n* (%)]	56 (71.8%)	18 (52.9%)	0.433	[−1.3; 39.1]	18.9%
Age [years, mean ± SD]	41.2 ± 10	38.5 ± 11	0.393	NA	2.7
Sex [female, *n* (%)]	48 (61.5%)	25 (73.5%)	0.265	[−12.0; −30.9]	−12.0%
Duration of visual symptoms ≥ 1 year [*n* (%)]	53 (67.9%)	20 (58.8%)	0.476	[−11.0; 29.3]	9.1%

Displayed are *p*-values, 95% confidence intervals (CIs) for the group differences in proportions, and the absolute group differences in percentage points. Group difference [%] was calculated as [% in pwMS with prior diagnostic work-up] – ([% in pwMS without prior diagnostic work-up]. The 95% CI refers to the absolute difference in proportions between the two groups. NA: not applicable for continuous variables (e.g. age); pwMS: people with multiple sclerosis.

**Figure 3.**
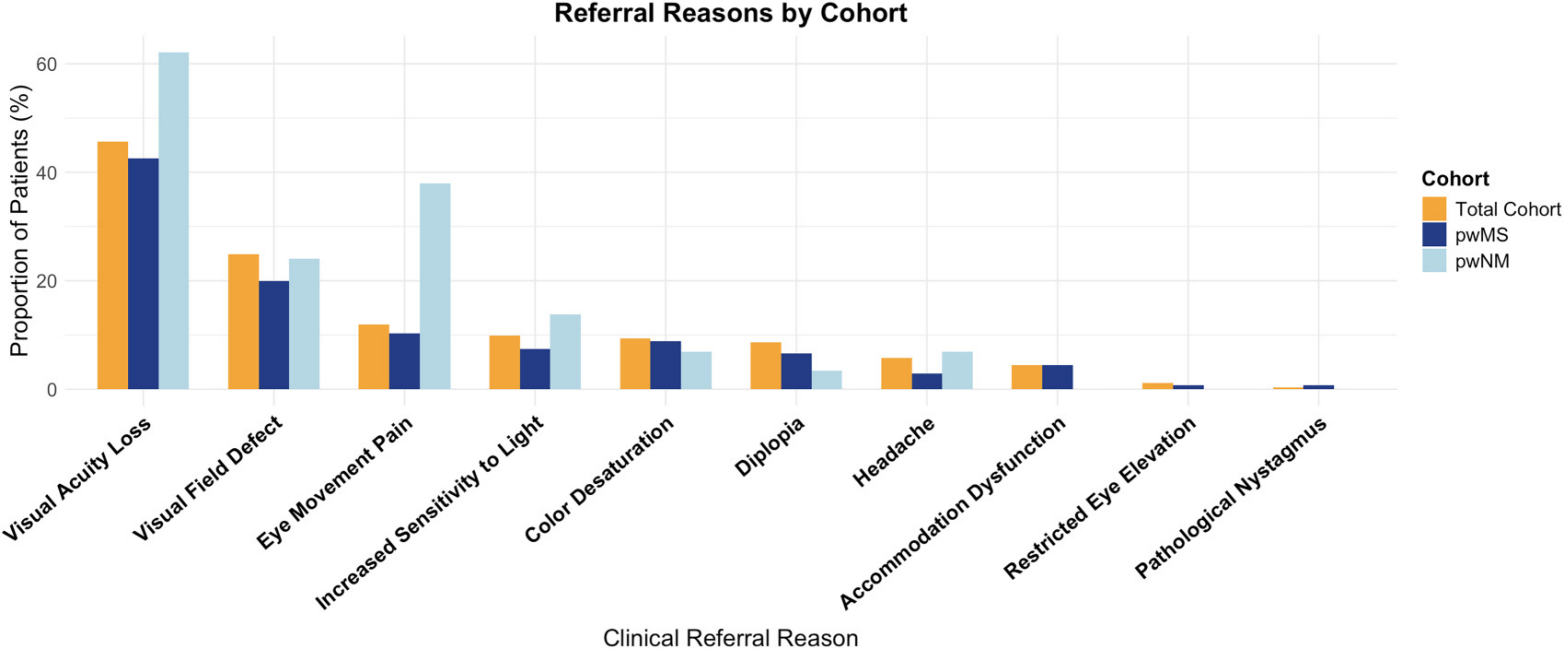
Referral reason to the neurovisual outpatient clinic by diagnostic subgroup. Bar plot showing the proportion of patients reporting different visual complaints at referral, stratified by diagnostic group (total cohort, pwMS, and pwNM). Visual acuity loss was the most frequent reason for referral across all groups. Percentages refer to the proportion of patients within each diagnostic subgroup who reported the respective symptom as part of the referral indication. pwMS: people with multiple sclerosis; pwNM: people with neuromyelitis optica spectrum disorders and myelin oligodendrocyte glycoprotein antibody-associated disease.

The frequency of prior diagnostic procedures did not differ between pwMS and pwNM for cMRI (*p* = 0.375, group difference = −8.7%), lumbar puncture (*p* = 0.719, group difference = −10.8%), and visual field testing (*p* = 0.665, group difference = 1.7%). Yet, blood samples (*p* < 0.001, group difference = –10.8%), VEP (*p* = 0.005, group difference = –17%) and OCT (*p* = 0.027, group difference = –10.5%) were more frequent in pwNM compared to pwMS. Referral Reasons to the neurovisual outpatient clinic are summarized in Figure 3.

**Figure 4.**
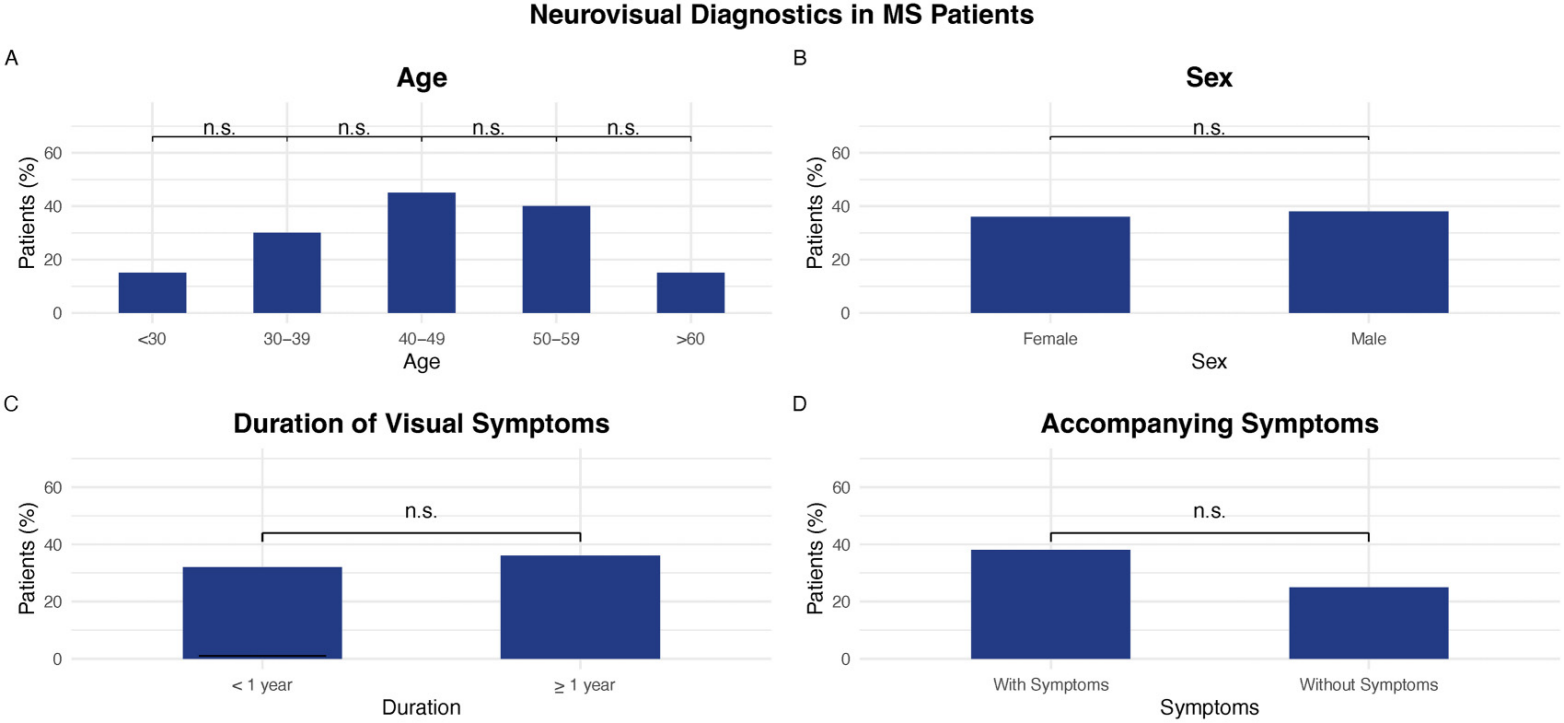
Utilization of specific neurovisual diagnostics (OCT, VEP and visual field) in pwMS does not differ in distinct demographic groups, including pwMS grouped by (a) age, (b) sex, (c) duration of visual symptoms, and (d) accompanying symptoms.

OCT: optical coherence tomography; VEP: visual evoked potentials; pwMS: people with multiple sclerosis; pwNM: people with neuromyelitis optica spectrum disorders and myelin oligodendrocyte glycoprotein antibody-associated disease.

Of the 112 pwMS analyzed, 78 patients (69.6%) had previously undergone diagnostic procedures, whereas 34 patients (30.4%) had not (Table 3). No group differences were observed regarding age (*p* = 0.393, group difference = 2.7%), sex distribution (*p* = 0.265, group difference = 12.0%), presence of accompanying symptoms (*p* = 0.433, group difference = 18.9%) or duration of visual symptoms over one year (*p* = 0.476, group difference = 9.1%). We also tested if barriers of care in pwMS with neurovisual symptoms depend on demographics: although pwMS aged 30–60 (Figure 4(A), *p* = 0.749) and pwMS with accompanying symptoms (Figure 4(D), *p* = 0.085) received numerically more diagnostic workup, these differences did not achieve statistical significance. Further, no differences in the frequency of diagnostic procedures were observed based on duration of visual symptoms (Figure 4(C), *p* = 0.474) or sex (Figure 4(B), *p* = 0.313). Exploratory stratification of specific diagnostics by time period (2015–2019 vs. 2020–2023) did not reveal significant differences in uptake (data not shown).

## Discussion

Employing outpatient data from an expert center in Germany, this study reveals a diagnostic imbalance between standard procedures and targeted specific diagnostics in pwMS with visual symptoms. Pre-referral OCT use was low (11.1% overall; 6.2% in pwMS), while cMRI, lumbar puncture and laboratory tests were substantially more frequent.

The utilization of diagnostic procedures was not associated with demographic or clinical variables, including age, sex, duration of visual symptoms, or the presence of accompanying symptoms. Although pwMS with accompanying symptoms received numerically more pre-referral diagnostics, these differences were not statistically significant, suggesting that heterogeneous non-visual complaints do not systematically influence diagnostic utilization. In practice, OCT, VEP and visual field testing are ordered when clinical signs indicate visual pathway involvement rather than for demographic reasons. Because these tests add value in both acute and chronic diseases, simple associations with symptom duration are not expected. When comparing pwMS to pwNM as disease controls, prior OCT and VEP were numerically higher in pwNM, plausibly reflecting more acute or severe visual presentations, yet OCT and visual field testing remained infrequent overall.

Especially, OCT stands out as a highly sensitive and objective procedure that allows quantitative detection of damage to the retina and optic nerve and whose clinical relevance in MS and NMOSD is now well established.^22–24^ Nevertheless, it was the least frequently used procedure prior to referral. In clinical practice, OCT findings can (i) document prior optic nerve injury and quantify residual structural loss after suspected optic neuritis, (ii) support the differential diagnosis and guide further tests, (iii) provide an objective baseline for monitoring and patient counselling.^22,25^ Moreover, quantitative OCT metrics — particularly peripapillary retinal nerve fiber layer (pRNFL) and ganglion cell-inner plexiform layer (GCIPL) thickness show predictive value for subsequent visual outcomes and disease progression, as demonstrated in our cohort studies.^26^ In Germany, the lack of reimbursement by public health insurance for pwMS might contribute to the underuse of OCT. Yet, coverage and access may vary across care settings and the results should be interpreted with caution, as we did not collect reimbursement data. In addition, a lack of knowledge and familiarity about the added diagnostic value of OCT in non-specialized facilities could also play a role. In contrast, VEP is an established component of neurophysiological diagnostics, is already routinely used in the context of several neurological disorders and is included as a standardized element in the training curriculum for neurology residents.^27^ In outpatient care, the high acquisition costs of OCT equipment and the lack of reimbursement within the German health insurance system constitute a significant barrier to implementation. Due to these limitations, referring neurologists might rely on ophthalmological consultations or specialized clinics for an extensive neurovisual assessment. From a future perspective, multicenter studies confirm a role for system-level mechanisms, which would support the establishment of improved referral pathways and multidisciplinary care structures. Such pathways should include targeted education on the clinical use of specific diagnostics, capacity building in non-specialist settings, and structured neurology–ophthalmology coordination to ensure timely and accurate care for patients with visual impairment.

With the inclusion of the optic nerve as a fifth anatomical region for demonstrating dissemination in space in the revised 2024 McDonald criteria, the clinical and diagnostic significance of neurovisual manifestations in pwMS has been notably elevated.^12,13^ Because our dataset (2015–2023) predates this revision, we report descriptive pre-referral utilization rather than adherence. Our data indicate that this re-evaluation has yet to be integrated into routine clinical practice. Specific diagnostic tests for the objective detection of optic nerve involvement were performed less frequently than more general neurological standard procedures. Although specific diagnostics may not be clinically actionable in all cases because other criteria may be sufficient to establish an MS diagnosis. This indicates structural barriers to access to these procedures—whether due to limited equipment, a lack of interdisciplinary coordination, or a lack of standardization of diagnostic pathways. Notably, even among pwMS with documented neurovisual symptoms, few had prior VEP or OCT. While the revision of the 2024 McDonald criteria emphasizes the role of optic nerve involvement in initial diagnosis, targeted specific diagnostics may also have a role in long-term disease monitoring and prognostication. Future work should evaluate whether and how integrating specific diagnostics into routine care improves diagnostic timeliness, accuracy, or patient-reported outcomes.

Several limitations of this study must be considered: Firstly, the retrospective study design limits the possibility of inferring causal relationships and may have led to an underreporting of diagnostic measures. The selection of data related to visual symptoms was made by the person who examined the patients and took their medical history. It must also be considered that pwMS undergo regular cMRI as part of routine diagnostics. We recorded only whether cMRI was performed pre-referral, not why. Thus, routine surveillance cMRI, common in pwMS, may inflate the recorded frequency and overstate differences versus specific diagnostics. Because indication, local capacity, and reimbursement were not collected, appropriateness cannot always be determined. The pattern may reflect a mix of appropriate triage or deferral to tertiary testing, routine MRI surveillance, operator constraints, and variability in provider familiarity. Secondly, the data originate from a specialized tertiary care center and the number of subjects in the disease-control group was low, which may limit the transferability of the results to other contexts. Our estimates, therefore, describe pre-tertiary utilization prior to referral and are not directly generalizable to primary or secondary care or other health systems. Additionally, it is important to recognize a potential referral bias: The selective referral pattern may have impacted the cohort composition and the observed rates of diagnostic utilization. The infrequent performance of basic procedures, such as visual field testing or VEP, prior to referral — irrespective of whether the referral originated from neurology or ophthalmology — still indicates the presence of systemic barriers within clinical care pathways. Also, not all visual problems in pwMS are captured by OCT, VEP, or visual field testing and self-reported complaints may reflect higher-order visuoperceptual factors. In this study, OCT, VEP, and visual fields serve as examples of specific diagnostics. They inform diagnosis, monitoring, and referral, but their findings are interpreted within the overall clinical assessment.

Thus, future studies should address these limitations through prospective, multi-center designs including expert and non-expert centers with the inclusion of patient-reported outcomes, time-to-diagnosis parameters and health economic analyses to better understand the described barriers to care.^28–30^ In addition, it would seem sensible to investigate how specific diagnostics such as OCT can be more efficiently embedded in diagnostic pathways — particularly in primary and secondary care — as part of healthcare research.^30,31^ Health policy measures, such as adjusted reimbursement or targeted training for referring physicians, could also make a decisive contribution to improving diagnostic fairness.^31^

In conclusion, this study provides real-world evidence of a diagnostic imbalance in pwMS with visual symptoms, illustrating both the limited integration of specific diagnostics and the variability of current care pathways. These findings may inform future prospective and health services research aimed at optimizing the use of OCT, VEP and visual field testing in routine clinical care.

## Supplemental Material

sj-docx-1-mso-10.1177_20552173251397772 - Supplemental material for Barriers to care for people with unclear visual loss—Data from a tertiary-level-of-care neuroinflammation center Supplemental material, sj-docx-1-mso-10.1177_20552173251397772 for Barriers to care for people with unclear visual loss—Data from a tertiary-level-of-care neuroinflammation center by Murat Delikaya, Charlotte Bereuter, Jan Schroeter, Elisa Nowak, Eva-Maria Dorsch, Lidia Kilinska, Joseph Kuchling, Nadja Siebert, Janina Behrens, Friedemann Paul, Judith Bellmann-Strobl, Tanja Schmitz-Hübsch and Frederike Cosima Oertel in Multiple Sclerosis Journal – Experimental, Translational and Clinical

## Data Availability

The data that support the findings of this study are not publicly available due to institutional and data protection policies and the category of institutional ethical approval.
