# Supplementary material for: Barriers to care for people with unclear visual loss—Data from a tertiary-level-of-care neuroinflammation center
Source: Mult Scler J Exp Transl Clin. 2025 Dec 5;11(4):20552173251397772. doi: 10.1177/20552173251397772 (PMC12681669; doi:10.1177/20552173251397772)
Supplement: sj-docx-1-mso-10.1177_20552173251397772 - Supplemental material for Barriers to care for people with unclear visual loss—Data from a tertiary-level-of-care neuroinflammation center [file sj-docx-1-mso-10.1177_20552173251397772.docx]

|  |  | Total Cohort | | | Multiple Sclerosis | | | NMOSD/MOGAD | | |
| --- | --- | --- | --- | --- | --- | --- | --- | --- | --- | --- |
| Diagnostic Test 1 | Diagnostic Test 2 | p | 95%-CI | Group Difference [%] | p | 95%-CI | Group Difference [%] | p | 95%-CI | Group Difference [%] |
| Blood Sample | cMRI | <0.001 | [-41.566, -26.688] | -34.13 | <0.001 | [-49.725, -22.07] | -35.9 | 0.762 | [-13.949, 26.449] | 6.25 |
|  | Lumbar Puncture | 0.209 | [-14.609, 2.705] | -5.95 | 0.148 | [-28.337, 2.696] | -12.82 | 0.101 | [0.139, 43.611] | 21.88 |
| cMRI | Lumbar Puncture | <0.001 | [20.806, 35.543] | 28.17 | 0.002 | [9.426, 36.728] | 23.08 | 0.088 | [-7.061, 38.311] | 15.62 |
| OCT | VEP | <0.001 | [-43.191, -28.238] | -35.71 | <0.001 | [-51.23, -25.693] | -38.46 | 0.005 | [-59.371, -15.629] | -37.5 |
|  | Visual Field | 0.413 | [-2.912, 8.468] | 2.78 | 0.764 | [-5.789, 10.918] | 2.56 | 0.109 | [0.819, 30.431] | 15.62 |
| VEP | Visual Field | <0.001 | [31.234, 45.75] | 38.49 | <0.001 | [28.683, 53.369] | 41.03 | <0.001 | [34.91, 71.34] | 53.12 |

**Supplementary Table** The group difference refers to the proportion of patients who received the respective diagnostic modality prior to presentation in the neurovisual clinic, calculated as the percentage for [Diagnostic **Test 1] – [**Diagnostic **Test 2]**. This comparison was performed separately for the total cohort, patients with multiple sclerosis (MS), and patients with NMOSD/MOGAD. A two-sided chi-square test was used to assess whether the difference in prior diagnostic frequency was statistically significant.
